# Human rabies in Côte d'Ivoire 2014-2016: Results following reinforcements to rabies surveillance

**DOI:** 10.1371/journal.pntd.0006649

**Published:** 2018-09-06

**Authors:** Issaka Tiembré, Anaïs Broban, Joseph Bénié, Mathilde Tetchi, Sophie Druelles, Maïna L’Azou

**Affiliations:** 1 Anti-rabies Center, National Institute of Public Hygiene, Abidjan, Côte d’Ivoire; 2 Global Epidemiology, Sanofi Pasteur, Istanbul, Turkey; 3 Global Epidemiology, Sanofi Pasteur, Lyon, France; Wistar Institute, UNITED STATES

## Abstract

In Côte d’Ivoire, rabies is endemic and remains largely uncontrolled. The numbers of human exposures and rabies cases are unknown and are probably much higher than reported. Data on human rabies cases are collected by the National Institute of Public Health (NIPH) Anti-rabies Center in Abidjan through a network of 28 NIPH local units, which cover the population of the entire country. During 2014, the NIPH initiated a program to reinforce the human rabies surveillance system in those 28 NIPH local units, with specific goals of improving the infrastructure, training, communication, and government involvement. Here, we report the progress and findings during 2014–2016. The reinforced system recorded 50 cases of human rabies (15–18 cases/year; annual incidence = 0.06−0.08 per 100,000) and more than 30,000 animal exposures (annual incidence = 41.8−48.0 per 100,000). Almost one-half of the human rabies cases were in children ≤15 years old. All were fatal and dog bites were the most common route by which rabies virus was transmitted. In the 32 cases where samples of sufficient quality for analysis were available, rabies was confirmed by reverse transcription-polymerase chain reaction RT-PCR. Post-exposure prophylaxis with rabies vaccine was administered to all animal exposure victims presenting at the NIPH local units, although only about 57% completed the full immunization schedule. All available reports were provided by the NIPH local units, indicating effective communication between them and the NIPH Anti-rabies Center. These findings indicate that the reinforcements resulted in highly specific detection of human rabies, provided detailed epidemiological data about these cases, and improved estimates of animal exposure numbers. These represent substantial advances, but further improvements to the surveillance system are needed to increase disease awareness and capture cases that are currently missed by the system. In the future, better communication between local health centers and the NIPH units, surveillance at the local health center level, and increased veterinarian engagement will help provide a more complete picture of the rabies burden in Côte d’Ivoire.

## Author summary

Rabies is a fatal viral disease. Transmitted mostly by dog bites, human rabies continues to be a substantial public health problem, especially in poorer countries. In Côte d’Ivoire, rabies is common and remains largely uncontrolled, but the true number of human rabies cases is unknown, so the magnitude of the problem remains unclear, complicating efforts to control this zoonosis. Data on human rabies cases in Côte d’Ivoire are collected by the National Institute of Public Health through a network of 28 NIPH local units. These NIPH local units provide rabies vaccine to people that may have been exposed to rabies virus. During 2014, the NIPH initiated a program to reinforce the rabies surveillance system in those NIPH local units, with specific goals of improving the infrastructure, training, communication and government involvement. Here, we describe the results of these efforts during 2014–2016. The reinforced surveillance system yielded detailed data about animal exposures and human rabies cases. This study confirmed the suspicion that many people who receive rabies prophylaxis do not finish the full set of vaccinations, putting them at unnecessary risk. Thus, the reinforcements represent an advance, but further improvements to the surveillance system are needed.

## Introduction

Rabies is a fatal viral infection, transmitted mostly by dog bites, that continues to be a substantial public health problem, especially in developing nations [1,2]. The most recent estimate, based on the number of dog bites and the probability of infection, is 59,000 human rabies deaths per year, most of which are in Africa and Asia [1].

In developing nations, rabies control has been especially hindered by a “vicious cycle of indifference and lack of information” [3]. The situation is worsened by insufficient infrastructure, along with the lack of an affordable high-quality vaccine [4,5]. Mass vaccination of dogs has proven effective in controlling rabies in many countries. The World Health Organization (WHO) has set a goal of eliminating dog-mediated rabies by 2030, but it has not been pursued in many developing nations [5]. Instead, human rabies is often managed by post-exposure prophylaxis (PEP), which includes washing bites or scratches and administering rabies vaccine with or without rabies immune globulin (RIG) to prevent a fulminant rabies virus infection [2]. In general, PEP has greatly reduced human rabies cases, but full compliance with the required multi-dose vaccination regimens and RIG has been poor [3].

In Côte d’Ivoire, as in most of Africa, rabies is endemic and remains largely uncontrolled, principally because less than 1% of the dogs are vaccinated against rabies [1,3]. In addition, the burden of the disease is not known. Data on human rabies in Côte d’Ivoire are collected by the National Institute of Public Health (NIPH) through a national network of regional and departmental units. At these NIPH local units, victims of WHO category II animal exposures (nibbling of uncovered skin, minor scratches or abrasions) and category III animal exposures (transdermal bites or scratches, licks on broken skin, contamination of mucous membrane with saliva from licks and exposure to bats) can receive PEP in the form of rabies vaccine only, as RIG is not available in Côte d’Ivoire. Due to the endemicity of rabies, the uncertain health status of the animals involved and the limited availability of veterinary resources to investigate animal exposures, all such exposures presenting at local health centers (LHC) are referred for PEP at the nearest NIPH unit.

Surveillance by the basic NIPH network, however, has been passive, inefficient, and not systematic. Case definitions have been inconsistent, healthcare providers have lacked adequate training and awareness, samples have not been routinely collected and analyzed, different forms have been used to collect data and data have been transmitted from NIPH local units to the central unit only once yearly. Therefore, the real numbers of animal exposures and rabies cases in Côte d’Ivoire are unknown and are probably much higher than reported by the NIPH in previous years. For example, the NIPH network detected 26 cases of human rabies between 2001 and 2009, only 4 of which were laboratory confirmed [6]. In contrast, Hampson et al. estimated 569 (95% confidence interval, 298–1352) cases of human rabies per year. This figure was based on the estimated number of animal bites nationwide and the probabilities that animals are infected, that rabies develops in the absence of PEP that PEP is sought, completed and successful [1]. To obtain a realistic view of the rabies problem in Côte d’Ivoire, more detailed and reliable data are needed.

During 2014, the NIPH initiated a national program to reinforce the existing rabies surveillance system in Côte d’Ivoire through the NIPH local units. Specific goals of the project were to improve infrastructure, training, communication of animal exposures and human cases, sampling and laboratory confirmation of human cases, and government involvement. Here, we describe the results of these efforts and the data obtained during 2014–2016.

## Materials and methods

### Study design

This was a descriptive prospective observational study performed in Côte d’Ivoire between 2014 and 2016. The objective of the study was to describe progress in making improvements to the Côte d’Ivoire’s rabies surveillance system. In addition, the incidence and characteristics of reported animal exposures and human rabies cases are reported.

### Ethics statement

This was a national public health surveillance activity sponsored by the NIPH and approved by the Ministry of Health and HIV Control of Côte d’Ivoire. Approval by an institutional review board or written informed consent from patients was not required. Data concerning the exposed humans and animal owners were anonymized before analysis.

### The NIPH human rabies surveillance system in Côte d’Ivoire

Epidemiological data on PEP for rabies and rabies cases is collected by 28 local NIPH units. All data from these NIPH local units are compiled by the NIPH Anti-rabies Center in Abidjan, Côte d’Ivoire. These NIPH local units are also responsible for patient care and, when possible, collecting tissue samples from suspected rabies human cases. Individuals potentially exposed to rabies virus through WHO category II and III animal exposures, such as victims of dog bites or other animal exposures, who seek treatment at any of the 82 LHC are to be referred to the nearest NIPH local unit for PEP with rabies vaccine only (RIG is not available in Côte d’Ivoire), administered by the intramuscular route. Suspected human rabies cases are also referred to the NIPH units for diagnosis and treatment[NB: what treatment do human rabies cases receive?]. However, it is important to note that this referral is voluntary and patients are not required to abide by these recommendations. In principle, data on animal exposures and rabies cases are to be collected at the NIPH units from individuals seeking PEP and then sent for analysis to the NIPH Anti-rabies Center in Abidjan. Any samples received at the Anti-rabies Center are to be transferred to the Pasteur Institute of Côte d’Ivoire (IPCI) to confirm rabies virus infection.

### Surveillance reinforcement measures

**Fig 1:**
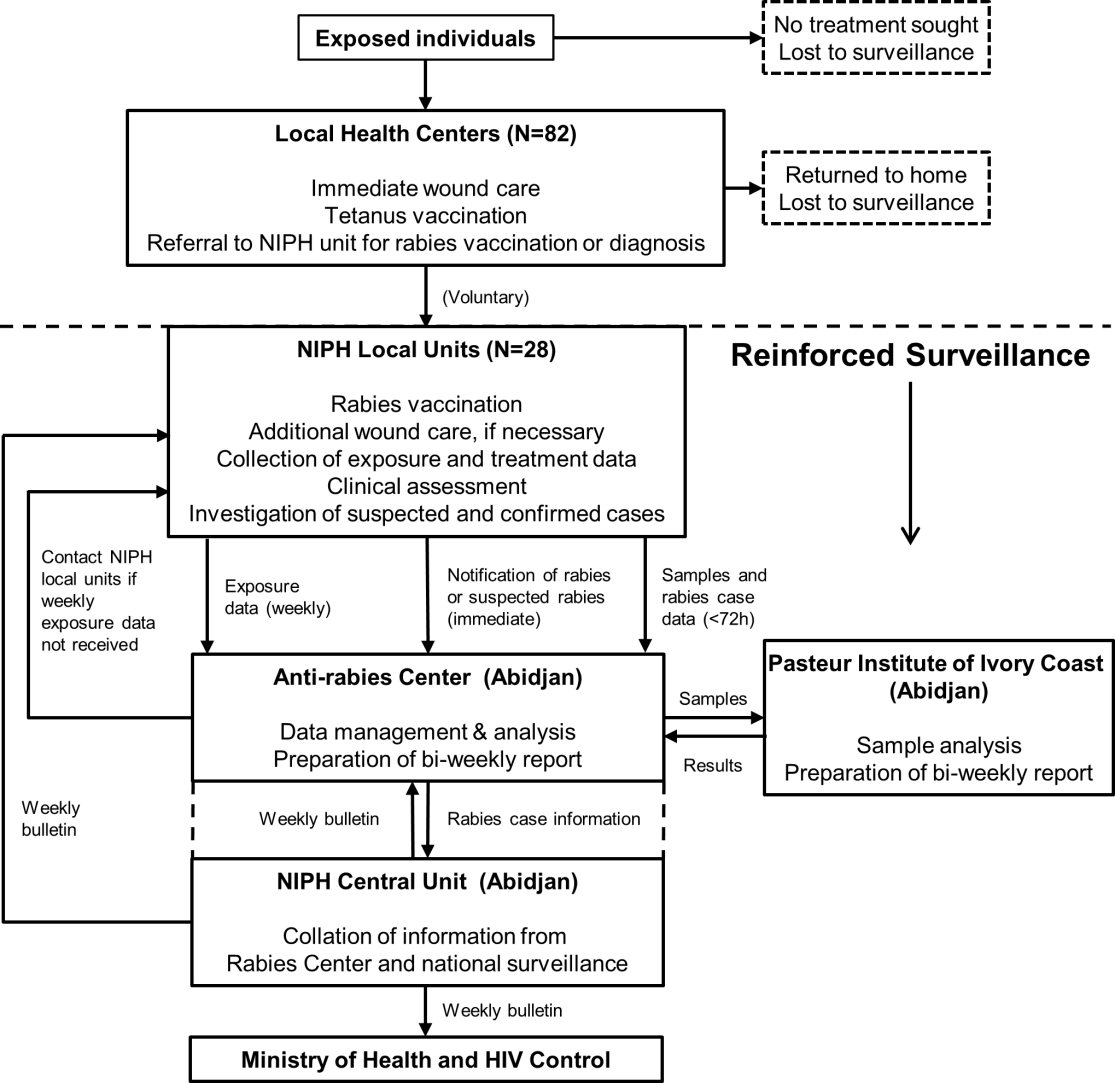
The reinforced National Institute of Public Health (NIPH) rabies surveillance network in Côte d’Ivoire. Exposed individuals presenting at local health centers received immediate wound care and were referred to a NIPH local unit for PEP with rabies vaccine. Suspected human rabies cases were also referred to the nearest NIPH local unit for diagnosis and treatment. Exposed individuals could also present directly at a NIPH local unit or at the Anti-rabies Center in Abidjan. Individuals choosing to present at a NIPH local unit or the Anti-rabies Center received rabies vaccine and additional wound care as necessary. At the 28 NIPH local units, animal exposure data were collected from the individuals presenting there and were transmitted weekly to the Anti-rabies Center located within the NIPH Central Unit in Abidjan. NIPH units that did not comply were systematically contacted by the Anti-rabies Center to collect the information. The Anti-rabies Center was immediately notified about clinically diagnosed or suspected cases of human rabies. In addition, samples and information about the case were collected and shipped to the Anti-rabies Center through the rural bus system as an unaccompanied package, which was retrieved by Anti-rabies Center personnel in Abidjan and directly transferred to the Pasteur Institute of Côte d’Ivoire, also in Abidjan. The Anti-rabies Center collaborated with the Pasteur Institute for laboratory confirmation of rabies virus by RT-PCR [NB: give a citation for the specific sop or provide a brief, accurate description]. Rabies case information from the surveillance network was transmitted to the NIPH Central unit where it was combined with other national surveillance data and disseminated in weekly bulletins to the Anti-rabies Center, NIPH local units, and the Ministry of Health and HIV Control, as well as national veterinary authorities, and district and national health authorities.

The surveillance system and its reinforcements are shown in **Fig 1**. In 2014, the existing NIPH surveillance system at 28 NIPH local units, covering the country population was reinforced by the addition of computers at the Anti-rabies Center in Abidjan, telephones and faxes for 10 of the NIPH local units that lacked them, and by supplying new kits to all 28 NIPH local units for collecting, preserving, and transporting skin and saliva samples from human rabies cases. In addition, 293 agents from these NIPH local units, 24 health district representatives, 27 agents from veterinary authorities, and 22 local representatives from community and local health authorities received a training manual and 2 to 3 hours of on-site standardized training. Training was provided by a multidisciplinary team and included the following: human rabies case recognition and definitions; types and severity of animal exposures; rabies surveillance network procedures and case notification; and procedures for taking, storing, and shipping samples. Furthermore, supervisors at each of the NIPH local units were briefed about the reinforcement project. Veterinarians were included to make them aware of the project and to assist in investigations of human rabies cases. At each NIPH local unit, two agents were vaccinated against rabies and were designated to collect data and samples from human rabies cases. During the second and third years of the study (2015 and 2016), supervision visits were made to each NIPH local unit to remind agents and supervisors about procedures, to discuss any needed changes, and to provide new kits for collecting, preserving and transporting tissue samples.

### Data and sample collection by NIPH local units during the study period

Individuals potentially exposed to rabies virus through category II and III animal exposures, and presenting at a LHC or NIPH local unit had any wounds cleaned and may have received the tetanus vaccine according to their vaccination status and willingness to be vaccinated (**Fig 1**). Because the rabies status of animals in Côte d’Ivoire is unknown in virtually all instances and further information on animal status is rarely available, all such exposures are considered potential exposures to rabies virus. In all such cases, the LHCs were to refer exposed individuals to a NIPH local unit for the full course of PEP. Suspected and probable human rabies cases presenting at a LHC were also to be referred to a NIPH local unit for diagnosis and treatment.

Animal exposure victims choosing to present at the NIPH local unit for PEP received Verorab (Sanofi Pasteur) by intramuscular injection according to the Zagreb (recommended; four doses in three visits) or Essen regimen (five doses in five visits) [7]. The regimen used depended on whether the patient chose to receive, or could afford, two doses (Zagreb) or one dose (Essen) of vaccine at the first visit. Additional wound care (e.g. additional cleaning and antibiotics) was administered as necessary. One of the two designated agents at the NIPH local unit who had been vaccinated against rabies collected the following data using a standardized form: patient demographics, characteristics of the animal exposure, details about the animal involved, and the treatments administered. Each week, data on animal exposures were compiled in a report and sent by the following Tuesday to the NIPH Anti-rabies Center at Abidjan by email, text message, phone, or fax. NIPH local units that did not comply were systematically contacted by the NIPH Anti-rabies Center to collect the information.

Human rabies was diagnosed clinically at NIPH local units based on the WHO definition, namely, presence of an acute neurological syndrome, characterized by forms of hyperactivity (furious rabies) or paralytic syndromes (paralytic rabies), developing to coma or death within 7 to 10 days after the first onset of symptoms [8]. Suspected and probable cases of rabies were identified by the following clinical signs and symptoms: headache, neck pain, nausea, fever, hydrophobia, anxiety, and agitation. Probable cases included a reliable history of animal exposure compatible with rabies virus transmission, and could also include abnormal tingling or pain at the site of the bite. In the event of a suspected or probable case of rabies, the NIPH Anti-rabies Center was notified immediately by one of the two designated agents via email, phone, text message, or fax. The designated agents were also to collect a skin biopsy from the nape of the neck, three saliva samples, and two urine samples, and to record the following data on the standardized form: samples taken, patient demographics, type of exposure, clinical signs, and information about the animal. Forms and samples (on ice) from suspected and probable cases were shipped to the NIPH Anti-rabies Center through the rural bus system as an unaccompanied package, which was retrieved by Anti-rabies Center personnel in Abidjan as soon as possible and transferred directly to the Côte d’Ivoire Pasteur Institute in Abidjan within a maximum total delay of 72 h from sample collection.

Findings on animal exposures and rabies cases were communicated via a weekly bulletin back to the NIPH local units, the Ministry of Health and HIV Control, national veterinary authorities, and district and national health authorities.

### Data management and analysis

Data from the NIPH local units were collected using Access (Microsoft, Redmond, WA, USA) and analyzed at the NIPH Anti-rabies Center using Epi Info software (US Centers for Disease Control and Prevention, Atlanta, GA, USA). For the statistical analysis of rabies cases, both clinically diagnosed and suspected rabies were included. Only descriptive analysis was performed. Missing data were not replaced. Catchment area populations refer to the combined populations of the several sanitary districts assigned to each of the 28 NIPH local units. These are defined by the government and the details are not available to the public. However, they were derived from administrative district populations obtained from the Côte d’Ivoire 2014 General Population and Housing Census [9].

### Confirmation of rabies virus infection

Samples were transferred from the NIPH Anti-rabies Center to the Côte d’Ivoire Pasteur Institute in Abidjan for confirmation of rabies virus infection by a nested RT-PCR, as described previously [10].

## Results

### Compliance with weekly reporting by NIPH local units

Of a possible 2968 weekly reports, all were received by the NIPH Anti-rabies Center. Of these, 2731 (92%) were delivered on time. For the remaining 8%, the NIPH local unit had to be contacted by the NIPH Anti-rabies Center.

### Reports of animal exposures

Between 2014 and 2016, a total of 32,411 animal exposures were reported by the 28 NIPH local units (**Table 1**). Annual animal exposure rates rose slightly each year from 41.8 per 100,000 in 2014 to 48.0 per 100,000 in 2016. The mean annual exposure rate was 45.2 per 100,000. By site, mean exposure rates ranged from a low of 19.3 per 100,000 in Dimbokro to a high of 122.5 per 100,000 at the Anti-rabies Center in Treichville.

**Table 1:** Number and incidence of animal exposures by region and overall reported by NIPH local units (2014–2016).

	Catchment area population	Number of exposures reported				Annual incidence of exposures per 100,000			
NIPH local unit		2014	2015	2016	Total	2014	2015	2016	Mean
Treichville	1,727,420	2320	2087	1940	6347	134.3	120.8	112.3	122.5
Port Bouet	978,726	281	497	523	1301	28.7	50.8	53.4	44.3
Yopougon	2,983,898	743	821	915	2479	24.9	27.5	30.7	27.7
Abengourou	580,391	334	273	268	875	57.5	47.0	46.2	50.3
Agboville	1,031,077	565	589	580	1734	54.8	57.1	56.3	56.1
Bondoukou	767,959	190	296	434	920	24.7	38.5	56.5	39.9
Bouaké	1,193,011	729	766	908	2403	61.1	64.2	76.1	67.1
Bouna	262,893	67	101	74	242	25.5	38.4	28.1	30.7
Boundiali	240,319	86	97	83	266	35.8	40.4	34.5	36.9
Dimbokro	932,199	189	165	185	539	20.3	17.7	19.8	19.3
Divo	1,002,723	325	370	395	1090	32.4	36.9	39.4	36.2
Ferkéssedougou	259,402	143	158	185	486	55.1	60.9	71.3	62.5
Gagnoa	798,308	425	432	403	1260	53.2	54.1	50.5	52.6
Guiglo	747,958	162	216	183	561	21.7	28.9	24.5	25.0
Katiola	395,885	96	145	127	368	24.2	36.6	32.1	31.0
Korhogo	610,844	342	374	414	1130	56.0	61.2	67.8	61.7
Man	1,377,035	574	675	719	1968	41.7	49.0	52.2	47.6
Odienné	447,097	147	174	172	493	32.9	38.9	38.5	36.8
Ouangolodougou	162,905	56	42	41	139	34.4	25.8	25.2	28.4
Tengrela	93,583	25	26	23	74	26.7	27.8	24.6	26.4
Touba	204,770	40	46	41	127	19.5	22.5	20.0	20.7
Yamoussoukro	1,222,346	272	361	387	1020	22.3	29.5	31.7	27.8
Aboisso	474,176	204	272	222	698	43.0	57.4	46.8	49.1
Daloa	1,836,975	598	629	582	1809	32.6	34.2	31.7	32.8
Séguéla	513,819	158	142	133	433	30.8	27.6	25.9	28.1
San Pedro	1,017,997	368	367	401	1136	36.1	36.1	39.4	37.2
Soubré	1,033,187	225	228	232	685	21.8	22.1	22.5	22.1
Abobo	1,030,658	349	568	911	1828	33.9	55.1	88.4	59.1
**Total**	**23,927,561**	**10,013**	**10,917**	**11,481**	**32,411**	**41.8**	**45.6**	**48.0**	**45.2**

Animal exposures (category II or III) were the numbers of exposed individuals presenting at each NIPH local unit for PEP. Catchment area populations refer to the combined population of the sanitary districts assigned to each of the 28 NIPH local units. These are defined by the government and derived from administrative district populations obtained from the Côte d’Ivoire 2014 General Population and Housing Census [9]. ARC, Anti-rabies Center.

In most of the cases (79%), the animal exposure site received primary or additional thorough cleansing at the NIPH local unit, and in 13% of the cases, tetanus vaccine was given (**Table 2**). Rabies PEP included active immunization against rabies in all cases. For most cases (81.5%), the Zagreb immunization schedule was used. However, only 56.6% of patients completed the full vaccination schedule.

**Table 2:** Animal exposure characteristics and treatments administered (2014–2016).

Characteristic	n (%)
Total animal exposures	32,411 (100.0)
**Treatments administered**	
Thorough cleansing of the wound	25,616 (79.0)
Tetanus vaccine administered	4,341 (13.4)
Active immunization with anti-rabies vaccine	32,411 (100.0)
Zagreb schedule	26,425 (81.5)
Essen schedule	5,986 (18.5)
Completed immunization schedule	18,329 (56.6)

Animal exposures were the numbers of exposed individuals presenting at a NIPH local unit for PEP.

### Human rabies cases

**Fig 2:**
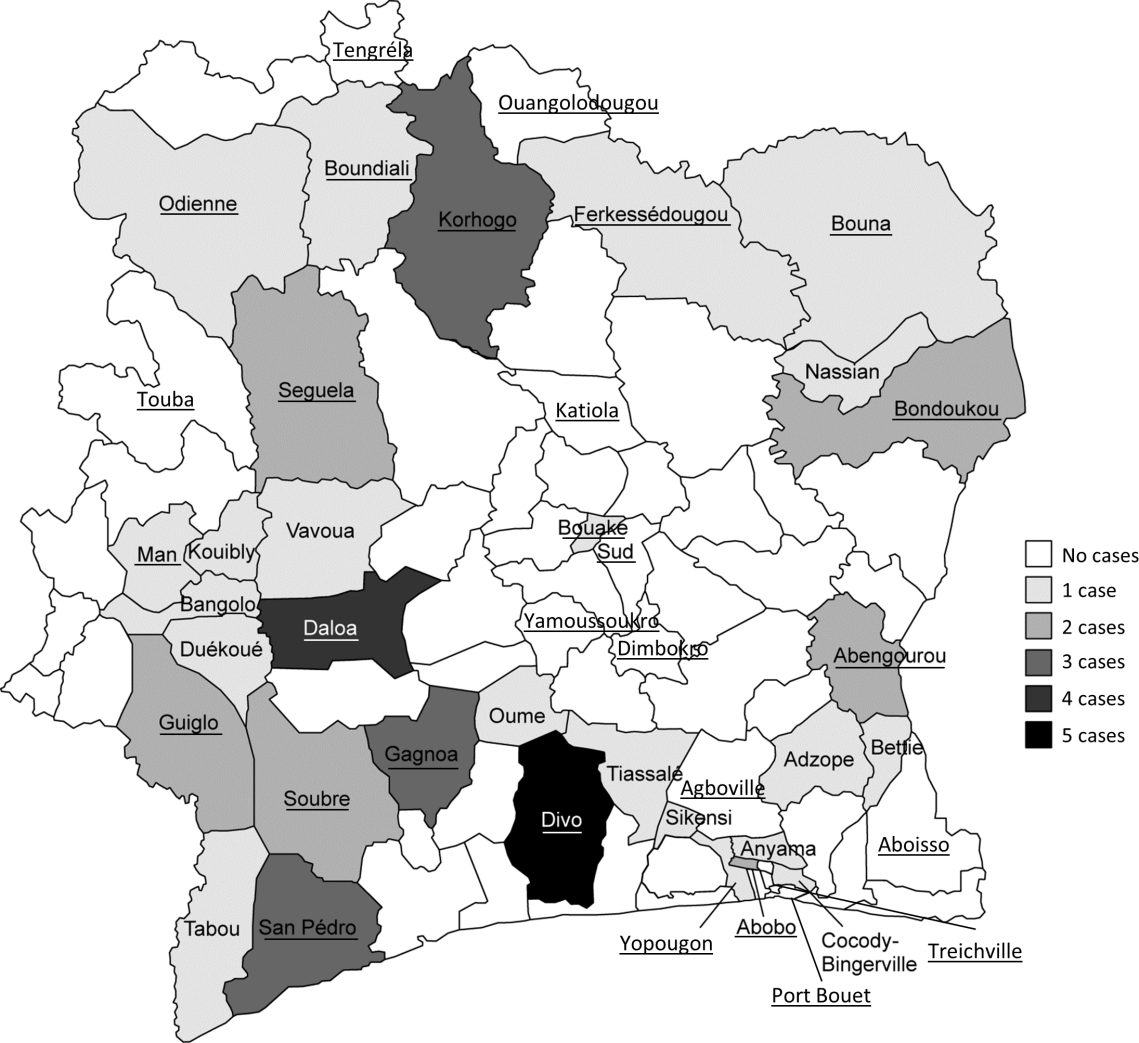
NIPH units and numbers of suspected or probable human rabies cases by region in Côte d’Ivoire during 2014–2016. Numbers of suspected or probable human rabies cases reported by the reinforced surveillance system are shown by the districts in which they occurred. Districts with NIPH units are shown in underlined text. Districts without names or shading had no rabies cases that were recorded by the surveillance program.

Fifty suspected or probable cases of human rabies, all fatal, were recorded, just under half of which (46%) were individuals under 15 years of age (**Table 3**). In all of these cases, the individuals already had clinical signs of rabies before they presented for the first time at a NIPH center. None had received PEP. Samples were available for testing in 39 cases and analyzable in 32 cases. In all 32 of these cases, rabies was confirmed by laboratory analysis. In most cases (96%), the exposure was a bite, and in most cases the animal involved was a dog (96%). In two-thirds of the cases, the exposure occurred in rural areas, where ~50% of the population lives [9]. The number of rabies cases reported per year ranged from 15 in 2014 to 18 in 2016. Accordingly, estimated annual incidence rates were between 0.06 and 0.08 per 100,000. The highest numbers of human rabies cases were reported in the regions of Divo (n = 5), Daloa (n = 4), Gagnoa, Korhogo, and San Pedro (n = 3 each) (**Fig 2**).

**Table 3:** Characteristics of suspected and probable human rabies cases in Côte d’Ivoire (2014–2016).

Characteristic	Rabies cases, n (%)
Total cases	50 (100)
Total cases reported per year, n [incidence per 100,000]	
2014	15 [0.06]
2015	17 [0.07]
2016	18 [0.08]
Type of rabies	
Furious	49 (98)
Paralytic	1 (2)
Sex	
Male	28 (56)
Female	22 (44)
Age group	
0–5 y	8 (16)
6–15 y	15 (30)
>15 y	27 (54)
Occupation	
Housekeeper	10 (20)
Student	14 (28)
None/unemployed	6 (12)
Farmer	9 (18)
Independent professional	3 (6)
Other	2 (4)
Missing	6 (12)
Biting animal	
Dog	48 (96)
Cat	2 (4)
Type of exposure	
Bite	48 (96)
Scratch	2 (4)
Location	
Rural	33 (66)
Urban	17 (34)
Treatments received at exposure	
Washing	5 (10)
Tetanus vaccine	7 (14)
Laboratory confirmation	
Sample not available	11 (22)
Sample quality insufficient for testing	7 (14)
Sample available and quality sufficient for testing	32 (64)
Positive for rabies	32 (64)

## Discussion

In 2014, NIPH initiated a program to reinforce the rabies surveillance system in Côte d’Ivoire. Specific goals of the project were to improve infrastructure, training, communication, and government involvement via dissemination of reports and information on human rabies. Thanks to these reinforcements, during 2014–2016, the system provided detailed surveillance data about exposures and human rabies cases. For all clinically suspected or probable cases of rabies with samples of sufficient quality for analysis, rabies was confirmed by RT-PCR. {NB: which samples were positive?] This is unusual in rabies-endemic countries, where it is rare to have quality samples for analysis and few human cases are confirmed [2]. Therefore, this represents an important advance. Moreover, it confirmed that the clinical assessment at the NIPH local units was appropriate and highly specific. During the three years of the study, all weekly reports from the NIPH local units were eventually transmitted to the NIPH Anti-rabies Center. Most (92%) were transmitted without reminders, confirming effective communication within the surveillance network. These are important steps forward because they mean that the surveillance system is providing detailed and timely data on animal exposures and rabies cases captured by the system, which should help obtain a clearer picture of human rabies in Côte d’Ivoire.

PEP was administered to all exposed individuals presenting at a NIPH local unit. As recommended by WHO guidelines, PEP included wound care, or additional wound care when necessary, and active immunization with rabies vaccine (but without the use of RIG). As found previously in Côte d’Ivoire [11,12], just over half of individuals who started PEP completed it. This poor compliance appears to be due to the lengthy, multiple-dose regimens, inaccessibility of treatment centers, and, possibly, the cost of PEP [3,11,13]. The government subsidizes 30% of the cost of rabies vaccine, but patients must still pay 8000 CFA francs (~14 USD) for each dose, which is a substantial sum for rural families.

During the three years of this study, the surveillance system recorded 50 cases of human rabies (15 to 18 cases annually) in Côte d’Ivoire. As found in other studies in Côte d’Ivoire, many of the cases were in children <15 years old and dog bites were the most common route of viral transmission [6,11,12]. None of these cases presented at a NIPH local unit prior to manifesting the clinical signs and none had received PEP. It is likely that these cases either never sought medical care or sought initial treatment at a LHC but did not seek further treatment or PEP at the NIPH local unit. Such cases illustrate the need to increase rabies awareness in communities, as most of these deaths could have been prevented with timely PEP following the exposures. In addition, these cases are evidence of a much larger pool of animal exposure cases that do not seek appropriate PEP within the healthcare system and therefore are currently not captured by surveillance. A previous study in Côte d’Ivoire recorded 26 national cases of human rabies in the 8½ years between January 2001 and June 2009 [6], which is lower than the 50 cases reported here for the entire population for the three years between 2014 to 2016. This suggests that the reporting within the reinforced NIPH surveillance system has improved over the system in previous years.

Although the reinforced network recorded over 32,000 animal exposures in 3 years, this number reflects only the persons who sought initial PEP at a NIPH local unit or who were referred to a NIPH unit by a LHC and chose to report there. Thus, it represents an undefined fraction of the total exposures that likely occurred within the catchment areas of the NIPH local units in the reinforced system. Persons not seeking follow-up treatment or any PEP at all would not be captured. Based on experience, we estimate that only 50−70% of the bite victims who seek treatment at a LHC actually report to a NIPH local unit to receive PEP. Many may not because PEP is too expensive or the NIPH unit is too far away. If the rough estimate of 50−70% for referral follow-up is accurate, the actual total number of animal exposure victims who sought initial treatment at LHCs may be closer to 14,045–21,067 per year on average, with a corresponding incidence of 58.7−88.0 per 100,000. However, some animal exposure cases seek initial treatment directly at a NIPH center rather than a LHC, so these adjusted estimates could be high. For comparison, Hampson et al estimated a bite incidence of 128 per 100,000 [1], which is between these adjusted estimates. The situation is similar for probable rabies cases presenting at a LHC for PEP who then do not present at the NIPH local unit after learning that no treatment can change the fatal outcome. However, we have no estimate for the referral success rate for rabies cases and all of these cases presented directly at a NIPH unit. Correction factors could be made more precise by collecting data at a representative number of LHCs to determine the number of animal exposure cases and rabies cases that seek PEP versus the number that actually report to a NIPH local unit, determining whether animal exposure cases presenting at a NIPH local unit sought initial treatment at a LHC. By performing community surveys, a better estimate may result for the overall numbers of animal exposures and rabies cases relative to those that seek healthcare treatment. These should be considered as the next steps to take in further strengthening the rabies surveillance system and could help establish relevant correction factors.

The numbers collected by the Côte d’Ivoire surveillance system are still substantially lower than other estimates of human rabies incidence that have been reported previously. Hampson et al. estimated 569 (95% confidence interval, 298–1352) cases per year and a per capita death rate of 2.64 per 100,000 based on an estimated 20,272 (95% confidence interval, 16,686–30,768) rabies virus exposures in 2015 [1]. The Global Burden of Disease project estimated 170 (95% confidence interval, 49–378) human rabies cases per year based on vital registration and verbal autopsy data from 2015 [14]. While such estimates could be inaccurate due to errors in underlying assumptions, the difference between these values and the numbers recorded by the Côte d’Ivoire surveillance system is not surprising. For a variety of reasons, surveillance systems usually underestimate the number of human rabies cases in rabies-endemic countries [15,16]. Human rabies cases are likely to be underreported in African countries. Inefficient and poorly structured surveillance systems lead to inadequate reporting and communication. Lack of enforcement prevents rabies cases to be properly reported to public health ministries. The lack of effective treatment results in affected individuals dying outside of the healthcare system [16,17]. In our study, even with improved surveillance and communication, the passive system was unable to capture animal exposures and rabies cases that did not present at a NIPH local unit.

The Hampson et al estimate of 20,272 annual rabies exposures is based on an estimated probability of 0.73 that a biting dog is rabid, which was used for many of the West African countries in their study [1]. This proportion is based on the number of animals found to be positive among animals suspected of being rabid that were submitted for laboratory analysis [18]. We were unable to confirm this proportion because very few animal diagnoses are performed in Côte d’Ivoire. If a similar proportion of the biting dogs in our study were indeed rabid, approximately 24,000 of the 32,411 animal exposures recorded over 3 years could be considered rabies virus exposures, with an annual incidence of 100.3 per 100,000. All of these individuals received at least some PEP and none were reported to have developed rabies. If the probability of developing rabies in the absence of PEP was between 0.02 and 0.19, as has been reported from studies in Tanzania [19,20], we estimate that between 480 and 4,560 rabies deaths were prevented by PEP within the scope of the reinforced system.

Animal exposures were consistently the most numerous, by a large margin, at the Anti-rabies Center in Treichville, resulting in a mean exposure incidence almost twice as high as any other NIPH unit. Located in the city of Abidjan, which is composed of ten municipalities, three of which have NIPH local units (Yopougon, Port-Bouet, and Abobo), the Treichville Anti-rabies Center was the first anti-rabies unit in Côte d’Ivoire and is the reference center in terms of patient care and epidemiological surveillance. As this the most well-known center, many animal exposure victims in the surrounding areas, with or without a NIHP unit, prefer to go directly to the Anti-rabies Center in Treichville.

Although enhanced surveillance can better define the problem, eliminating or reducing the number of human rabies cases in Côte d’Ivoire is the primary goal. Increasing the proportion of dogs vaccinated against rabies, which has been estimated to be less than 1% in Côte d’Ivoire [1], is the most efficient and cost-effective means of reducing viral transmission to humans. However, difficulties in obtaining governmental support and other resources necessary to implement a mass vaccination program make this a long-term solution that will take time to develop and achieve. In the immediate term, delivering effective PEP remains the principal means of reducing human rabies cases and should be a priority. As demonstrated in this study, poor patient compliance (56.6%) with the multi-dose regimens currently in use continues to be a problem that increases the risk of developing rabies after exposure. In Côte d’Ivoire, the most frequent reasons for not completing the immunization schedule have been financial, in that animal owners refused to pay for treatment or that patients were unable to pay for it, though other factors such as awareness and clinic proximity were also responsible [21]. Other impediments to compliance have been reported to include loss of wages, forgotten dates and the distance and time needed to travel to the clinic [13]. In principle, PEP compliance could be increased by: reducing patient costs by increasing the government subsidization above the current rate of 30%; requiring animal owners to pay for PEP; reducing travel expenses with shorter vaccination schedules requiring fewer clinic visits and making vaccine more widely available; and use of intradermal (ID) vaccinations that require smaller, more cost-effective volumes [22]. For example, alternative vaccination regimens have been proposed that can be completed within 1 week with three intramuscular vaccinations in two visits [23] or four ID vaccinations in three visits [24]. Current WHO guidelines recommend such ID PEP regimens, which are effective cost- and vaccine-saving measures that require up to 80% less vaccine per dose than intramuscular administration [25]. These alternative vaccination protocols should be investigated as ways to reduce costs and increase PEP compliance in Côte d’Ivoire.

In summary, the reinforcements made in 2014 have added to the reliability of the Côte d’Ivoire rabies surveillance system, while the extensive data collected will be important for increasing rabies awareness and for recruiting additional political support and resources for rabies prevention and control. In a similar surveillance reinforcement program in Cameroon, which was implemented in specific regions rather than nationally, the mean animal exposure incidence in the departments with surveillance reinforcement (26.4 per 100,000) was over fourfold higher than in those without it (6.1 per 100,000) [26]. Results from these efforts demonstrate that improved surveillance systems can provide evidence that the animal exposure and rabies virus exposure burdens in endemic areas in Africa can be much higher than those estimated from poorly functioning passive reporting systems. These data also provide a baseline that can be used to assess the impact of future rabies control efforts. The reinforcements represent some initial steps to improve the surveillance system, but further measures are needed. Immediate goals include improving communication and cooperation among the NIPH rabies surveillance network, LHCs, and public health authorities. In the future, improving engagement and collaboration with veterinary services would help to better define the burden of canine rabies and the numbers of rabies virus exposures. Finally, incorporating the LHCs into the reinforced surveillance system also would add another important level of surveillance that would help establish the frequency of successful referrals to NIPH local units. This is currently under consideration.

## Supporting information

S1 Data Raw data from Jan-Dec 2014. (PDF)

S2 Data Raw data from Jan-Dec 2015. (PDF)

S3 Data Raw data for Jan 2014-June 2016. (PDF)

S4 Data Rabies cases for 2016. (PDF)

S5 Data Complete raw data 2014–2016. (PDF)
