# Supplementary material for: Human rabies in Côte d'Ivoire 2014-2016: Results following reinforcements to rabies surveillance
Source: PLoS Negl Trop Dis. 2018 Sep 6;12(9):e0006649. doi: 10.1371/journal.pntd.0006649 (PMC6126804; doi:10.1371/journal.pntd.0006649)
Supplement: S1 Data — (PDF) [file pntd.0006649.s001.pdf]

INSTITUT NATIONAL D'HYGIENE PUBLIQUE

Projet de renforcement de la surveillance de la rage en côte d'Ivoire RAB42

Rapport Final

Présenté par Pr Ag Tiembré Issaka, Investigateur coordonateur

SANOFI PASTEUR

Liste des Abréviations

CAR : Centre Antirabique

INHP : Institut National d'Hygiène Publique

IPCI : Institut Pasteur de Côte d'Ivoire

SMIT : Service des Maladies infectieuses et Tropicales

Tableau I: Répartition des agents formés dans le cadre du projet RAB42 en fonction de la provenance

**Agents**	**Effectif**	**Pourcentage**
Agents INHP	293	80,05
Agents Districts sanitaires	22	6,01
Agents services Vétérinaires	27	7, 38
Agents services Mairie	24	6,56
**Total**	366	100

Tableau II : Données des cas de rage humaine de Janvier à décembre 2014, projet RAB 42

age	sexe	prof	date d'expo	Localité	lieu d'hospi	prélèvement d'échantillons	date de décès
*21	M	Electricien batiment	27/11/2013	Terre rouge (sanpedro)	néant	non	30-déc-13
*42	M	Planteur	fin nov 2013	Bouali (Daloa)	Maladies Infectieuses et Tropicales du CHU Treichville	non	26-janv-14
84	F	Ménagère	déc 2013	NZUEKRO (Bouaké)	CHU Bouaké	oui: 1,2 et 3	04-févr-14
4	F	ELEVE	janv 2014	Aboudoukro (Niablé)	CHR Abengourou	oui: 1,2 et 3	EVADEE
13	F	ELEVE	déc 2013	FRESCO	néant	oui: 1,2 et 3	14-mars-14
30	M	Manœuvre agricole	02/02/2014	VROUHO 2	CHR Ségula	oui: 1 et 2	02-avr-14
46	M	Planteur	fevrier 2014	DIGNANGO	néant	oui: 1 et 2	09-avr-14
41	M	Agent immobilier	fevrier 2014	YOPOUGON Gesco	CHU Treichville (SMIT)	oui: 1 et 3	17-avr-14
**30	F	Ménagère	1er avril 2014	Tahou (Kouibly)	néant	oui: 1 et 2	19-mai-14
9	M	Elève	06 Juillet 2014	Abengourou	CHR Abengourou	oui: 1 , 2 et 3	13 Aout 2014
22	M	Sans	19 Juin 2014	Iboklebe (Tabou)	CHR San - Pédro	oui: 2	17 Aout 2014
6	M	ELEVE	aout 2014	Séguela	CHR Séguela	oui: 1,2 et 3	21-oct-14

37	F	Ménagère	Octobre 2014	Sidama (Kong)	HG ferké	oui: 1 et 2	22-nov-14
**43	F	Ménagère	Septembre 2014	Bettié (M'batakro)	HG Béttié / CHR Abengourou	oui: 2	18-déc-14
34	F	Ménagère	Novembre 2014	gbeleban (Odienné)	CHR Odienné	oui: 1,2 et 3	encore vivante

** : Cas suspects non prélevés survenus avant la formation des agents*
** * : Prélèvements non conformes*

Figure 1 : Exposition à la rage humaine par mois de Janvier à décembre 2014, projet RAB 42

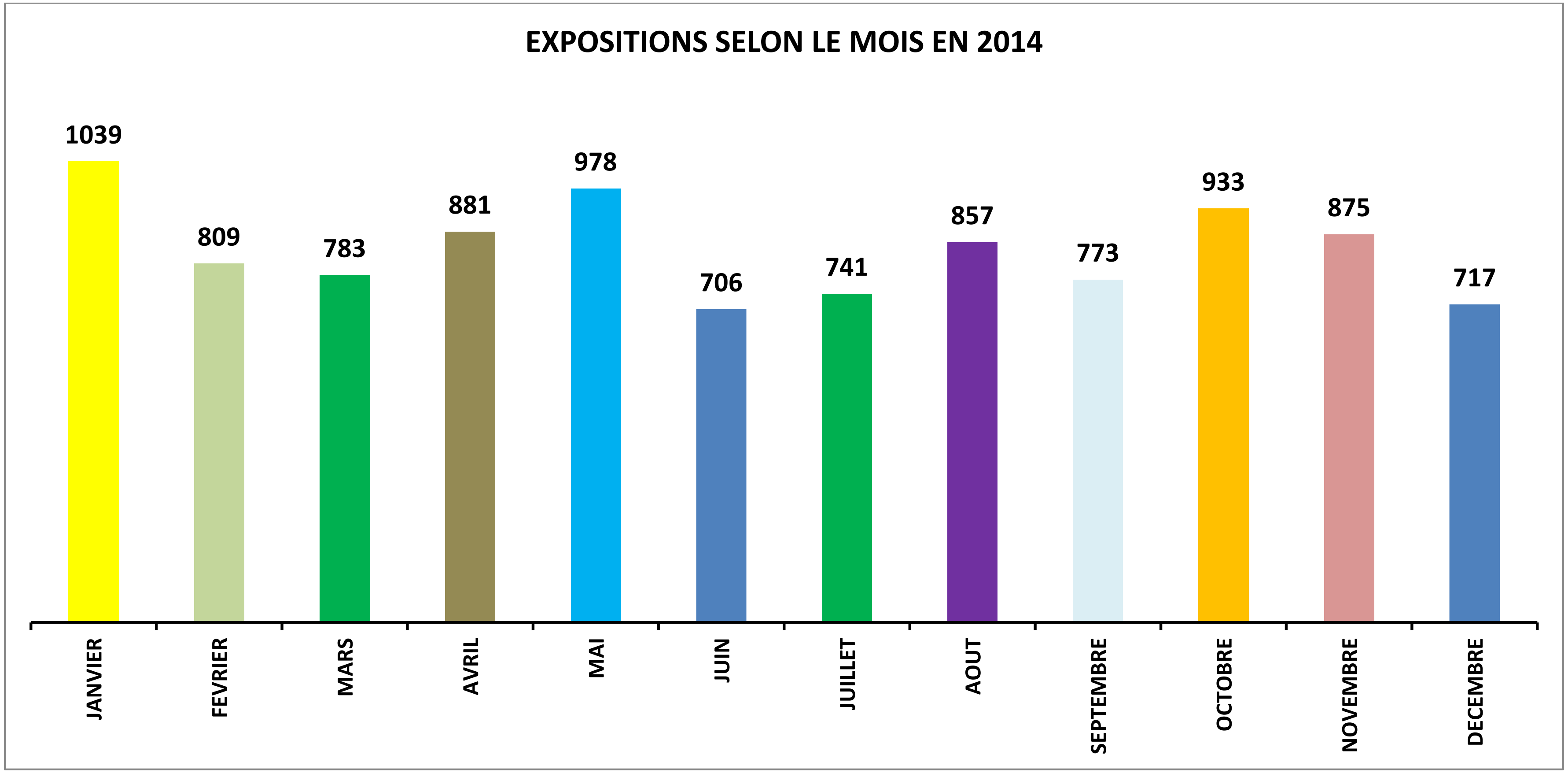

Figure 2 : Exposition à la rage humaine par antenne de Janvier à décembre 2014, projet RAB 42

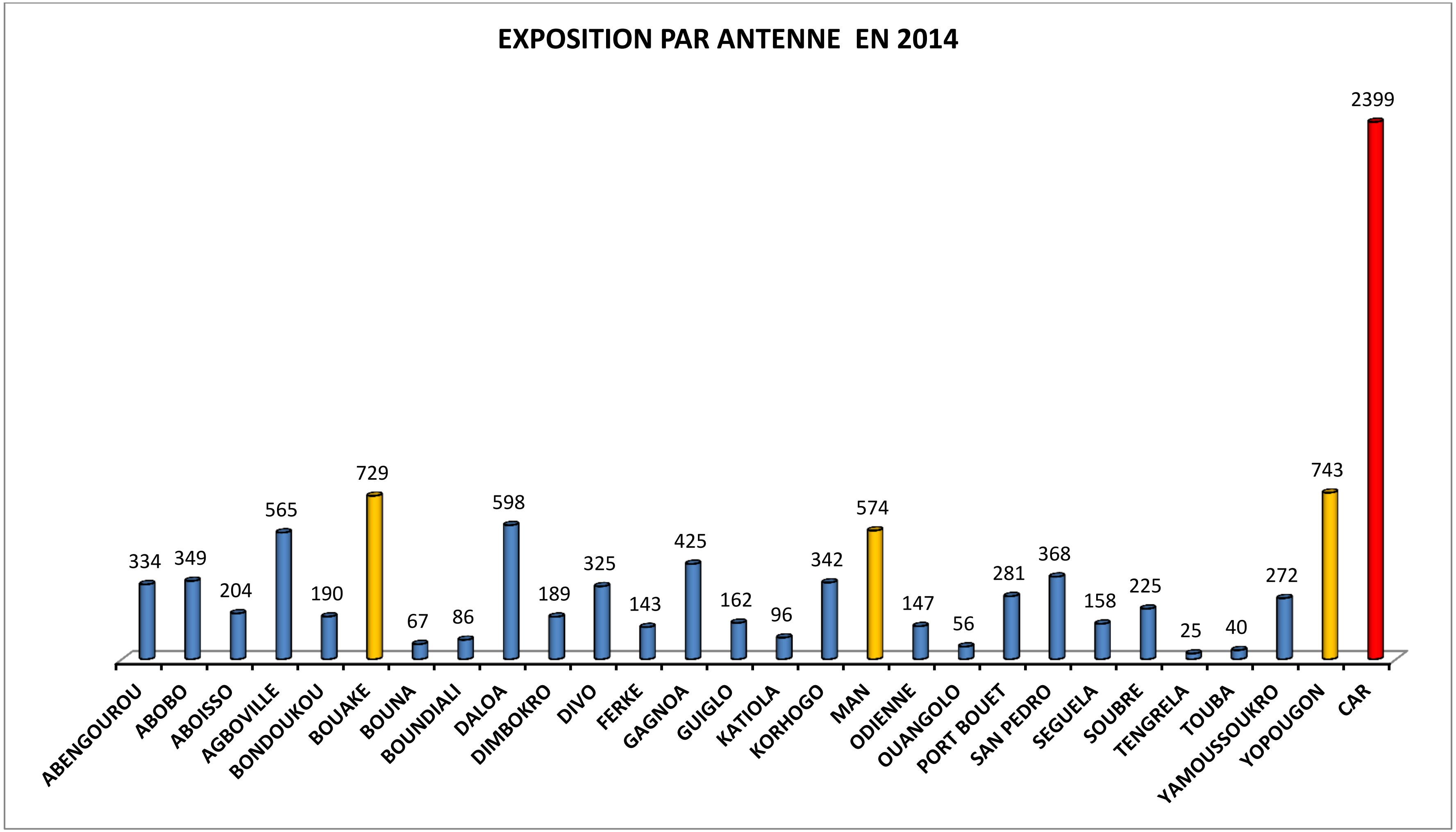

Tableau III : Cas d'exposition à la rage humaine en 2014 par âge et par antenne

ANTENNES	0-5 ANS	6-15 ANS	➢ 15 ANS	TOTAL
ABENGOUROU	**45**	136	153	**334**
ABOBO	**50**	152	147	**349**
ABOISSO	**23**	81	100	**204**
AGBOVILLE	**73**	211	281	**565**
BONDOUKOU	**26**	80	84	**190**
BOUAKE	**108**	299	322	**729**
BOUNA	**13**	8	46	**67**
BOUNDIALI	**11**	44	31	**86**
DALOA	**85**	270	243	**598**
DIMBOKRO	**23**	77	89	**189**
DIVO	**48**	122	155	**325**
FERKESSEDOUGOU	**20**	69	54	**143**
GAGNOA	**39**	161	225	**425**
GUILGLO	**21**	59	82	**162**
KATIOLA	**12**	34	50	**96**
KORHOGO	**55**	139	148	**342**
MAN	**97**	226	251	**574**
ODIENNE	**24**	60	63	**147**
OUANGOLO	**12**	31	13	**56**
PORT-BOUET	**51**	88	142	**281**
SAN PEDRO	**38**	168	162	**368**
SEGUELA	**33**	57	68	**158**
SOUBRE	**33**	97	95	**225**
TENGRELA	**5**	4	16	**25**
TOUBA	**4**	14	22	**40**
YAMOUSSOUKRO	**27**	109	136	**272**
YOPOUGON	**78**	304	361	**743**
CAR TREICHVILLE	**276**	**808**	**1315**	**2399**
**TOTAL**	**1330**	**3908**	**4854**	**10092**
