# Supplementary material for: Human rabies in Côte d'Ivoire 2014-2016: Results following reinforcements to rabies surveillance
Source: PLoS Negl Trop Dis. 2018 Sep 6;12(9):e0006649. doi: 10.1371/journal.pntd.0006649 (PMC6126804; doi:10.1371/journal.pntd.0006649)
Supplement: S2 Data — (PDF) [file pntd.0006649.s002.pdf]

**Projet de renforcement de la surveillance de la rage en Côte d'Ivoire RAB42**

Rapport préliminaire du projet – Janvier - décembre 2015

*Présenté par Professeur Tiembré Issaka, Investigateur coordonnateur*

*10 Aout 2016*

# I. Résultats

## II.1. Point sur les cas de rage humaine

### II.1.1. Qualité des prélèvements

Tableau I : Répartition des prélèvements selon la qualité, Projet RAB42, 2015

| Qualité du prélèvement | Effectif | Pourcentage |
|---|---|---|
| Non prélevés | **6** | **35,3** |
| Prélèvements Conformes | **5** | **29,4** |
| Prélèvements non Conformes | **6** | **35,3** |
| **Total** | **17** | **100** |

### II.1.2. Epidémiologie descriptive des cas

- **Répartition géographique des cas**

Au cours de l'année 2015, sur les dix-sept (17) cas suspects de rage humaine détectés 5 ont été confirmés par l'IPCI soit 29,4% dont la répartition est dans la figure 1.

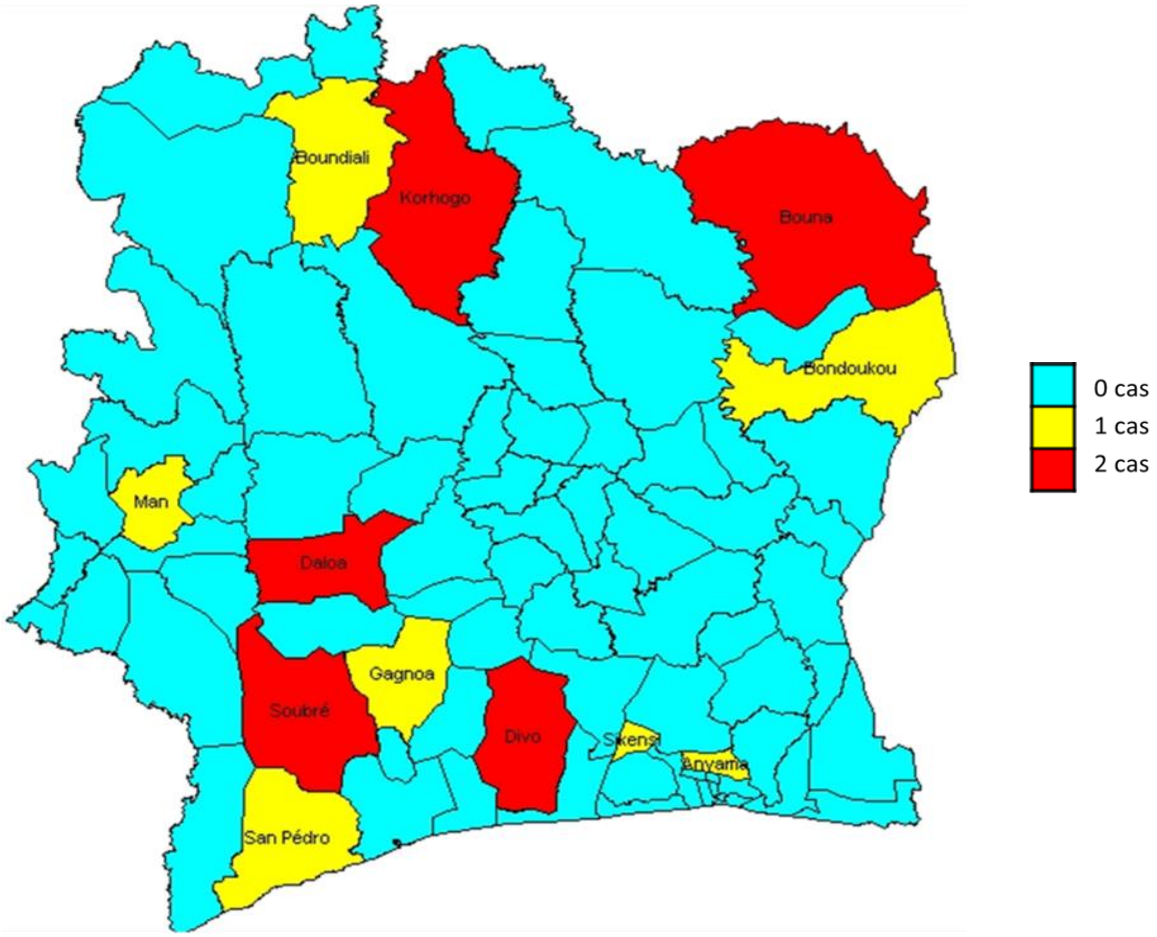


**Figure 1 : distribution des cas suspects de rage humaine, Cote d'Ivoire, 2015**

**Tableau II : Répartition des patients selon la profession**

| **Profession** | **Nombre** | **Pourcentage** |
|---|---|---|
| Sans profession | 6 | 35,30 |
| Ménagère | 5 | 29 ,40 |
| Elevé | 3 | 16,65 |
| Planteur ou cultivateur | 3 | 16,65 |
| **Total** | **17** | **100** |

**Tableau III : Répartition des personnes exposées à la rage, 2015 par tranche d'âge.**

| **Tranche d'âge** | **Nombre de cas d'exposition** |
|---|---|
| 0-5 ans | 1505 |
| 6-15 ans | 4348 |
| > 15 ans | 5064 |
| **Total** | **10 917** |

**Tableau IV : Données des cas de rage humaine de Janvier à décembre 2015, projet RAB 42**

| Semaine de Notification | Age (Année) | Sexe | Profession | Animal | Date expo | Localité | Prélèvement échantillons | date de décès | District sanitaire |
|---|---|---|---|---|---|---|---|---|---|
| 2 | 24 | F | Ménagère | chien | 01 /12/14 | Logoualé | oui: 1,2 et 3 | 10/01/15 | MAN* |
| 4 | 5 | M | Néant | chien | 25/12/14 | Koraleara (Korhogo) | oui: 2 et 3 | 23/01/15 | Korhogo* |
| 12 | 60 | F | Ménagère | chat | 26 /02/15 | Progreagui (Méagui) | oui: 1 | 24/03/15 | Soubré** |
| 13 | 9 | F | Néant | chien | 01/01.15 | Gadago (grand-zatri) | oui: 1 | 22/03/15 | Soubre** |
| 15 | 73 | M | Planteur | chien | 25/02/15 | Sikensi | Non | 11/04/15 | Sikensi*** |
| 15 | 60 | F | Ménagère | chat | 14 /03/15 | Anyama | Oui: 1 et 2 | 18/04/15 | Anyama* |
| 20 | 8 | M | élève | Chien | 08 /04/15l | Hiré | Non | 13/05/15 | DIVO*** |
| 21 | 34 | M | Planteur | Chien | 24 /02/15 | Gagnoa | Non | 24/05/15 | Gagnoa*** |
| 22 | 15 | M | élève | Chien | 01 /12/14 | San-pédro | Oui: 2 | 27/05/15 | San-Pédro*** |
| 25 | 17 | F | Ménagère | chiot | 13 /04/15 | Daloa | Oui: 1 et 2 | 21/06/15 | Daloa* |
| 31 | 4 | F | Néant | Chien | 15/07/15 | Divo | Oui: 1 et 2 | 24/07/15 | Divo** |
| 32 | 4 | M | Néant | Chien | inconnu | Boundiali | Oui: 2 | 03/08/15 | Boundiali* |
| 38 | 9 | M | élève | chien | 16 /08/15 | Gogobio | Oui: 1 et 3 | 20/09/15 | Daloa** |
| 43 | 19 | F | Ménagère | chien | 30 /09/15 | Wonanfanyon | Non Prélevé | 21/10/15 | Bouna*** |

| | | | | | | | | | |
|---|---|---|---|---|---|---|---|---|---|
| 48 | 4 | M | Sans | chien | 23 /10/15 | Dikodougou | Oui: 2 | 23/11/15 | Korhogo** |
| 49 | 32 | F | Cultivateur | Chien | 03 /11/15 | Bondoyo (Nassian) | Non Prélevé | 30/11/15 | Bouna*** |
| 50 | 5 | M | néant | Chien | 13 /11/15 | Abokro(Assuefry) | Oui: 1 et 3 | 19/12/15 | Bondoukou** |

Clés de couleur : cas confirmés par l'IPCI ; prélèvement non conforme ; cas non prélevés.

Clés d'échantillons : 1 = salive ; 2 = peau ; 3 = urines

F, femelle ; M, mâle; N ; non enregistré
