# Supplementary material for: Human rabies in Côte d'Ivoire 2014-2016: Results following reinforcements to rabies surveillance
Source: PLoS Negl Trop Dis. 2018 Sep 6;12(9):e0006649. doi: 10.1371/journal.pntd.0006649 (PMC6126804; doi:10.1371/journal.pntd.0006649)
Supplement: S3 Data — (PDF) [file pntd.0006649.s003.pdf]

Tableau1 : Nature de l'exposition

NaturExpo	Fréquence	Pourcentage
**Griffure**	2	5.30%
**Morsure**	36	94.70%
**Total**	38	100.00%

Tableau2 :**L'animal responsable**

Animal Responsable	Fréquence	Pourcentage
**Chien**	36	94.70%
**Chat**	2	5.30%
**Total**	38	100.00%

Tableau3 : **Provenance des cas**

Prov Cas	Fréquence	Pourcentage
**Village**	25	65.80%
**Ville**	13	34.20%
**Total**	38	100.00%

Tableau4 : **Lavage de la plaie**

LavPlaie	Fréquence	Pourcentage
**Oui**	4	10.50%
**Non**	34	89.50%
**Total**	38	100.00%

Tableau5 : **Administration de SAT**

Ad SAT	Fréquence	Pourcentage
**Oui**	7	18, 4%
**Non**	29	76, 3%
**Non précisé**	2	5,3%
**Total**	38	100.00%

Tableau6 : **Désinfection plaie**

DésinfPlaie	Fréquence	Pourcentage
**Oui**	7	18, 4%
**Non**	29	76, 3%
**Non précisé**	2	5,3%
**Total**	38	100.00%

Tableau7 : **Forme de rage**

RagFurieuse	Fréquence	Pourcentage
**Oui**	37	97.40%
**Non**	1	2.60%
**Total**	38	100.00%

Tableau8 : **Cas prelevés**

PrelevEchantillons	Fréquence	Pourcentage
**Oui**	28	73.70%
**Non**	10	26.30%
**Total**	38	100.00%

Tableau9 : **Cas Résultats d'analyses**

Résultats analyses	Fréquence	Pourcentage
**Confirmés**	22	78.60%
**Inadéquats**	6	21.40%
**Total**	28	100.00%

Tableau10 : **Post-exposure treatments**

Thorough cleansing of the wound = (lavage de la plaie)	Number of cases(nbre de cas)	Percentage(pourcentage)
Yes	21711	82%
No	4922	18%
Unknown = (ne sait pas)		
Anti-tetanic vaccine		
Yes	2875	11%
No	23758	89%
Unknown		
Anti-rabiesserum (immunoglobuline)		
Yes	0	
No	0	
Unknown		
Rabies vaccine schedule = (protocole antirabique)		
Yes	ZAGREB = 21340<br>ESSEN = 5293	80%<br>20%
If yes, finished vaccine schedule (respect du calendrier vaccinal)		
Yes	15147	57%
No	11486	43%
Unknown		
No		
Unknown		
TOTAL	26633	100%

Taux d'incidence :

INHP center	Population	Nb bites 2014	Nb bites 2015	Nb bites Jan-June 2016	Total	Incidence rate (/100 000) 2014	Incidence rate (/100 000) 2015	Estimated incidence rate (/100 000) 2016 *	Mean yearly incidence rate (/100 000) TOTAL
Treichville	1727420	2320	2087	931	5338	134.3	120.8	107.8	**123.6**
Port Bouet	978726	281	497	273	1051	28.7	50.8	55.8	**43.0**
Yopougon	2983898	743	821	486	2050	24.9	27.5	32.6	**27.5**
Abengourou	580391	334	273	149	756	57.5	47.0	51.3	**52.1**
Agboville	1031077	565	589	262	1416	54.8	57.1	50.8	**54.9**
Bondoukou	767959	190	296	230	716	24.7	38.5	59.9	**37.3**
Bouaké	1193011	729	766	458	1953	61.1	64.2	76.8	**65.5**
Bouna	262893	67	101	35	203	25.5	38.4	26.6	**30.9**
Boundiali	240319	86	97	45	228	35.8	40.4	37.5	**37.9**
Dimbokro	932199	189	165	101	455	20.3	17.7	21.7	**19.5**
Divo	1002723	325	370	216	911	32.4	36.9	43.1	**36.3**
Ferkéssedougou	259402	143	158	92	393	55.1	60.9	70.9	**60.6**
Gagnoa	798308	425	432	198	1055	53.2	54.1	49.6	**52.9**
Guiglo	747958	162	216	80	458	21.7	28.9	21.4	**24.5**
Katiola	395885	96	145	68	309	24.2	36.6	34.4	**31.2**
Korhogo	610844	342	374	221	937	56.0	61.2	72.4	**61.4**
Man	1377035	574	675	342	1591	41.7	49.0	49.7	**46.2**
Odienné	447097	147	174	98	419	32.9	38.9	43.8	**37.5**
Ouangolodougou	162905	56	42	20	118	34.4	25.8	24.6	**29.0**
Tengrela	93583	25	26	10	61	26.7	27.8	21.4	**26.1**

Touba	204770	40	46	22	108	19.5	22.5	21.5	**21.1**
Yamoussoukro	1222346	272	361	206	839	22.3	29.5	33.7	**27.5**
Aboisso	474176	204	272	106	582	43.0	57.4	44.7	**49.1**
Daloa	1836975	598	629	296	1523	32.6	34.2	32.2	**33.2**
Séguéla	513819	158	142	69	369	30.8	27.6	26.9	**28.7**
San Pedro	1017997	368	367	169	904	36.1	36.1	33.2	**35.5**
Soubré	1033187	225	228	112	565	21.8	22.1	21.7	**21.9**
Abobo	1030658	349	568	408	1325	33.9	55.1	79.2	**51.4**
**TOTAL**	**23927561**	**10013**	**10917**	**5703**	**26633**	**41.8**	**45.6**	**47.7**	**44.5**

Répartition géographique des cas

Tableau de répartition des cas par CAR INHP

CAR INHP	Nbre de cas 2014	Nbre de cas 2015	Nbre de cas Janv-Juin 2016	TOTAL
Abengourou	3	0	0	3
Adzope	0	0	1	1
Bondoukou	0	2	0	2
Bouake	1	0	0	1
Bouna	0	1	0	1
Boundiali	0	1	0	1
CAR Treichville	0	2	0	2
Daloa	1	2	0	3
Divo	0	2	1	3
Ferke	1	0	0	1
Gagnoa	2	1	2	5
Guiglo	0	0	1	1
Korhogo	0	2	0	2
Man	1	1	0	2
Odienne	1	0	0	1
San Pedro	2	1	0	3
Seguela	2	0	1	3
Soubre	0	2	0	2
Yopougon	1	0	0	1
TOTAL	15	17	6	38

Tableau de répartition des cas par District sanitaire

District Sanitaire	Nbre cas 2014	Nbre cas 2015	Nbre cas Janv-Juin 2016	TOTAL
**Abengourou**	2	0	0	2
**Adzope**	0	0	1	1
**Anyama**	0	1	0	1
**Bettie**	1	0	0	1
**Bondoukou**	0	1	0	1
**Bouake Sud**	1	0	0	1
**Bouna**	0	1	0	1
**Boundiali**	0	1	0	1

**Daloa**	1	2	0	3
**Divo**	1	2	1	4
**Ferke**	1	0	0	1
**Gagnoa**	1	1	1	3
**Guiglo**	0	0	1	1
**Korhogo**	0	2	0	2
**Kouibly**	1	0	0	1
**Man**	0	1	0	1
**Nassian**	0	1	0	1
**Odienne**	1	0	0	1
**Oume**	0	0	1	1
**San Pedro**	1	1	0	2
**Seguela**	2	0	0	2
**Sikensi**	0	1	0	1
**Soubre**	0	2	0	2
**Tabou**	1	0	0	1
**Vavoua**	0	0	1	1
**Yopougon Ouest**	1	0	0	1
**TOTAL**	15	17	6	38
