# Supplementary material for: Human rabies in Côte d'Ivoire 2014-2016: Results following reinforcements to rabies surveillance
Source: PLoS Negl Trop Dis. 2018 Sep 6;12(9):e0006649. doi: 10.1371/journal.pntd.0006649 (PMC6126804; doi:10.1371/journal.pntd.0006649)
Supplement: S4 Data — (PDF) [file pntd.0006649.s004.pdf]

Cas de la rage pour 2016

(Semaine Notification)	âge (ans)	sexe	profession	animal responsable	date d'exposition	Localité	prélèvement d'échantillons	date de décès	Distrist sanitaire
1	19	M	élève	chien	06 décembre 2015	Oumé	Oui: 1 et 3	09-janv-16	Oumé
1	6	F	Sans	chien	10 décembre 2015	Vavoua	Oui: 1,2 et 3	10-janv-16	Vaoua
6	7	F	élève	chien	21 novembre 2015	Zikisso	Non	10-févr-16	Gagnoa
7	10	M	élève	chien	janvier 2016	Fresco	Oui: 1,2 et 3	20-févr-16	Divo
8	13	M	élève	chien	Non précisé	Taî	Non	évadé	Guiglo
12	18	M	élève	chien	06 février 2016	Adzopé	Non	14-mars-16	Adzopé
29	13	F	Sans	chien	mai 2016	Bingerville	Oui: 1	21-juil-16	Cocody-Bingerville
30	4	M	Sans	chien	03 juillet 2016	Abobo	Oui: 1 et 3	30-juil-16	Abobo-Ouest
31	29	M	Planteur	chien	06 juillet 2016	Tiassalé (Bacanda)	Non	01 aout 2016	Tiassalé
32	35	M	Agent mairie	chien	juillet 2016	Gabiadji	Oui: 2	14 aout 2016	San Pedro
34	49	M	Cultivateur	chien	juin 2016	Tahoudi	Oui: 1 et 3	28 aout 2016	Bondoukou
34	3	F	Sans	Chiot	mai 2016	Tabou (Para)	Oui: 1 et 3	22 aout 2016	Tabou
35	11	M	élève	chien	25 juin 2016	Duekoué	Oui: 2	26 aout 2016	Duékoué
41	10	F	élève	chien	1er Sept 2016	Yabligué	Oui: 1 et 3	15-oct	Bangolo
44	46	M	Cultivateur	chien	03-sept-16	Katoumara	Oui: 1 et 3	08-nov	Korhogo
47	22	M	Agent de sécurité	chien	23 Aout 2016	Abobo	Oui: 1 et 3	26-nov-16	Abobo-Ouest
48	15	F	Elève	Chien	Aout 2016	Daloa (Brizéboua)	Oui: 2	30-nov-16	Daloa
49	65	M	Cultivateur	Chien	08-oct-16	Data (Divo)	Oui: 1 et 3	12-déc-16	Divo
