# Supplementary material for: Human rabies in Côte d'Ivoire 2014-2016: Results following reinforcements to rabies surveillance
Source: PLoS Negl Trop Dis. 2018 Sep 6;12(9):e0006649. doi: 10.1371/journal.pntd.0006649 (PMC6126804; doi:10.1371/journal.pntd.0006649)
Supplement: S5 Data — (PDF) [file pntd.0006649.s005.pdf]

Tableau 1 : Répartition des personnes exposées par antenne INHP (calcul d'incidence) 2014-2016

Centre d'INHP	Population	No. morsures 2014	No. morsures 2015	No. morsures 2016	Somme	Taux d'incidence (/100 000) 2014	Taux d'incidence (/100 000) 2015	Taux d'incidence (/100 000) 2016	Moyen taux d'incidence (/100 000) TOTAL
Treichville	1,727,420	2320	2087	1940	6347	134.3	120.8	112.3	122.5
Port Bouet	978,726	281	497	523	1301	28.7	50.8	53.4	44.3
Yopougon	2,983,898	743	821	915	2479	24.9	27.5	30.7	27.7
Abengourou	580,391	334	273	268	875	57.5	47.0	46.2	50.3
Agboville	1,031,077	565	589	580	1734	54.8	57.1	56.3	56.1
Bondoukou	767,959	190	296	434	920	24.7	38.5	56.5	39.9
Bouaké	1,193,011	729	766	908	2403	61.1	64.2	76.1	67.1
Bouna	262,893	67	101	74	242	25.5	38.4	28.1	30.7
Boundiali	240,319	86	97	83	266	35.8	40.4	34.5	36.9
Dimbokro	932,199	189	165	185	539	20.3	17.7	19.8	19.3
Divo	1,002,723	325	370	395	1090	32.4	36.9	39.4	36.2
Ferkéssedougou	259,402	143	158	185	486	55.1	60.9	71.3	62.5
Gagnoa	798,308	425	432	403	1260	53.2	54.1	50.5	52.6
Guiglo	747,958	162	216	183	561	21.7	28.9	24.5	25.0
Katiola	395,885	96	145	127	368	24.2	36.6	32.1	31.0
Korhogo	610,844	342	374	414	1130	56.0	61.2	67.8	61.7
Man	1,377,035	574	675	719	1968	41.7	49.0	52.2	47.6
Odienné	447,097	147	174	172	493	32.9	38.9	38.5	36.8
Ouangolodougou	162,905	56	42	41	139	34.4	25.8	25.2	28.4
Tengrela	93,583	25	26	23	74	26.7	27.8	24.6	26.4
Touba	204,770	40	46	41	127	19.5	22.5	20.0	20.7
Yamoussoukro	1,222,346	272	361	387	1020	22.3	29.5	31.7	27.8
Aboisso	474,176	204	272	222	698	43.0	57.4	46.8	49.1
Daloa	1,836,975	598	629	582	1809	32.6	34.2	31.7	32.8
Séguéla	513,819	158	142	133	433	30.8	27.6	25.9	28.1
San Pedro	1,017,997	368	367	401	1136	36.1	36.1	39.4	37.2

Soubré	1,033,187	225	228	232	685	21.8	22.1	22.5	22.1
Abobo	1,030,658	349	568	911	1828	33.9	55.1	88.4	59.1
**Somme**	**23,927,561**	**10,013**	**10,917**	**11,481**	**32,411**	**41.8**	**45.6**	**48.0**	**45.2**

Tableau 2 : Informations sur la prise en charge des personnes exposées

	Nombre de cas	Pourcentage
**Lavage de la plaie**		
**Oui**	25616	79%
**Non**	6795	21%
**Ne sait pas**		
**Vaccin anti-tetanique**		
**Oui**	4341	13%
**Non**	28070	87%
**Ne sait pas**		
**Immunoglobuline antirabique**		
**Oui**	0	
**Non**	0	
**Ne sait pas**		
**Protocole de vaccin antirabique**		
**Oui**	ZAGREB = 26425<br>ESSEN = 5986<br>TOTAL = 32411	82%<br>18%
**Si oui, respect du calendrier vaccinal?**		
**Oui**	18239	56%
**Non**	14172	44%
**Ne sait pas)**		
**Somme**	32411	100%

Résultats d'analyse des cas de rage enregistrés en Côte d'Ivoire de 2014 à 2016

Tableau 1 : Nature de l'exposition

Nature Exposition	Fréquence	Pourcentage
Morsure	48	96.00%
Griffure	2	4.00%
Léchage	0	0.00%
Somme	50	100.00%

Tableau 2 : animal responsable

Animal responsable	Fréquence	Pourcentage
Chien	48	96.00%
Chat	2	4.00%
Somme	50	100.00%

Tableau 3 : Provenance des cas

Localité	Fréquence	Pourcentage
Village	33	66.00%
Ville	17	34.00%
Somme	50	100.00%

Tableau 4 : Lavage des plaies

Lavage Plaie	Fréquence	Pourcentage
Oui	5	10.00%
Non	45	90.00%
Somme	50	100.00%

Tableau 5 : Injection contre tétanos

Injection de Sérum antitétanique	Fréquence	Pourcentage
Oui	7	14.90%
Non	40	85.10%
Inconnu	3	0.06%
Somme	50	100.00%

Tableau 6 : Prélèvement d'échantillons

Prélèvement Echantillons	Fréquence	Pourcentage
Oui	39	78.00%
Non	11	22.00%
Somme	50	100.00%

Tableau 7 : Résultat analyse échantillons

Forme Rage	Fréquence	Pourcentage
Rage Furieuse	49	98.00%
Rage Paralytique	1	2.00%
Somme	50	100.00%

Tableau 8 : Forme de rage

Résultat Laboratoire	Fréquence	Pourcentage
Confirmés	32	82.10%
Non traités	7	17.90%
Somme	39	100.00%

Tableau 9 : Répartition par sexe

Sexe	Fréquence	Pourcentage
F	22	44.00%
M	28	56.00%
Somme	50	100.00%

Tableau 10 : Répartition des cas par groupe d'âge

Age Groupe	Fréquence	Pourcentage
0 à 5 ans	8	16.00%
6 à 15 ans	15	30.00%
Plus de 15 ans	27	54.00%
Somme	50	100.00%

Tableau 11 : Répartition des groupes d'âge selon la provenance

Age Groupe	Village	Ville	TOTAL
0 à 5 ans	4	4	8
6 à 15 ans	8	7	15
Plus de 15 ans	21	6	27
Somme	33	17	50

Tableau 12 : cas prélevés en rapport avec la notion d'hospitalisation

	PRELEVECHANTILLONS		
Hospitalisation	Yes	No	TOTAL
Oui	31	7	38
Non	8	4	12
Somme	39	11	50

Tableau13 : Répartition des victimes selon la profession

Profession	Fréquence	Pourcentage
Agent sécurité	2	4.00%
Elève	14	28.00%
Menagère	10	20.00%
Neant (bas âge)	6	12.00%
Planteur	9	18.00%
Prof libérale	3	6.00%
Sans emploie	6	12.00%
Somme	50	100.00%

Tableau 14 : Notion de Vaccination Anti-rab chez les victimes

Vaccin Anti-Rab	Fréquence	Pourcentage
Oui	8	16.00%
Non	42	84.00%
Somme	50	100.00%

Tableau de distribution des cas de rage de 2014 à 2016 par district sanitaire

Districts sanitaires	Cas 2014	Cas 2015	Cas 2016	Somme
Abengourou	2	0	0	2
Abobo Ouest	0	0	2	2
Adzope	0	0	1	1
Anyama	0	1	0	1
Bangolo	0	0	1	1
Bettie	1	0	0	1
Bingerville	0	0	1	1
Bondoukou	0	1	1	2
Bouake	1	0	0	1
Bouna	0	1	0	1
Boundiali	0	1	0	1
Daloa	1	2	1	4
Divo	1	2	2	5
Duékoué	0	0	1	1
Ferkessédougou	1	0	0	1
Gagnoa	1	1	1	3
Guiglo	0	0	2	2
Korhogo	0	2	1	3
Kouibly	1	0	0	1
Man	0	1	0	1
Nassian	0	1	0	1
Odienne	1	0	0	1
Oume	0	0	1	1
San Pédro	1	1	1	3
Seguela	2	0	0	2
Sikensi	0	1	0	1
Soubre	0	2	0	2
Tabou	1	0	0	1
Tiassalé	0	0	1	1
Vavoua	0	0	1	1
Yopougon Ouest	1	0	0	1
TOTAL	15	17	18	50

Tableau de Notification des cas de rage humaine 2014 à 2016 par antenne INHP

Antenne	Cas 2014	Cas 2015	Cas 2016	Somme
Abengourou	3	0	0	3
Abobo	0	0	2	2
Adzope	0	0	1	1
Bondoukou	0	2	1	3
Bouake	1	0	0	1
Bouna	0	1	0	1
Boundiali	0	1	0	1
CAR Treichville	0	2	2	4
Daloa	1	2	1	4
Divo	0	2	2	4
Ferkessedougou	1	0	0	1
Gagnoa	2	1	2	5
Guiglo	0	0	4	4
Korhogo	0	2	1	3
Man	1	1	0	2
Odienne	1	0	0	1
San Pédro	2	1	1	4
Seguela	2	0	1	3
Soubre	0	2	0	2
Yopougon	1	0	0	1
**TOTAL**	**15**	**17**	**18**	**50**
